# Guideline-Based Statin Eligibility, Coronary Artery Stenosis and Cardiovascular Events in Patients with Stable Chest Pain: A Secondary Analysis of the PROMISE Randomized Clinical Trial

**DOI:** 10.3390/jcm9103076

**Published:** 2020-09-24

**Authors:** Amit Pursnani, Jana Taron, Thomas Mayrhofer, Michael T. Lu, Maros Ferencik, Joseph A. Ladapo, Pamela S. Douglas, Udo Hoffmann

**Affiliations:** 1 Cardiology Division, NorthShore University Health System, Evanston, IL 60201, USA; APursnani@northshore.org; 2 Cardiovascular Imaging Research Center, Department of Radiology, Massachusetts General Hospital, Harvard Medical School, Boston, MA 02114, USA; TMAYRHOFER@mgh.harvard.edu (T.M.); mlu@mgh.harvard.edu (M.T.L.); UHOFFMANN@mgh.harvard.edu (U.H.); 3 Department of Radiology, Freiburg University Hospital, 79106 Freiburg, Germany; 4 School of Business Studies, Stralsund University of Applied Sciences, 81435 Stralsund, Germany; 5 Knight Cardiovascular Institute, Oregon Health and Science University, Portland, OR 97239, USA; MFERENCIK@mgh.harvard.edu; 6 Division of General Internal Medicine and Health Services Research, David Geffen School of Medicine at UCLA, Los Angeles, CA 90024, USA; jladapo@mednet.ucla.edu; 7 Duke Clinical Research Institute, Duke University School of Medicine, Durham, NC 27708, USA; pamela.douglas@duke.edu

**Keywords:** 2018 ACC/AHA prevention guidelines, statin eligibility, computed tomography angiography, coronary artery disease

## Abstract

Background: Recommendations for preventive statin treatment in patients with stable chest pain may be difficult as symptoms can be unspecific. It is unclear if coronary CT angiography (CTA)-detected coronary artery disease (CAD) can optimize statin prescription. Methods: In stable chest pain patients randomized to CTA in the PROMISE trial, statin eligibility was defined per 2018 American College of Cardiology/American Heart Association (ACC/AHA) guidelines. Primary outcome was a composite of death, myocardial infarction or unstable angina over 26 months median follow-up. Hazard ratios (HR) of non-obstructive (1–69% stenosis) and obstructive (≥70% stenosis) CAD for events were determined using Cox proportional hazard models. Calculated HR were then incorporated into the ACC/AHA pooled cohort equation (PCE) to revised ASCVD risk and assess re-classification of statin eligibility. Results: Among 3986 patients (60.5 ± 8.2 years; 51% female), 72.9% (2904/3986) were statin eligible. Event rates in statin-eligible vs. ineligible patients were 3.3% vs. 2.3% (HR = 1.4 (95% CI 0.9–2.2), *p* = 0.142). Although the proportion of statin-eligible patients increased with CAD severity, 54% without CAD were statin eligible. Incorporating information on CAD into PCE reclassified 12.7% of patients (1.3% towards statin, 11.4% towards no statin). Similar results were found in stratified analysis of statin naïve patients (reclassification of 13.9%, 1.0% towards statin, and 12.9% towards no statin). As a result, revised ASCVD risk improved model discrimination in all patients (c-statistic: 0.59 (95 %CI 0.55–0.62) vs. 0.52 (95 %CI 0.49–0.56); *p* 0.001), while reducing statin use by 10.1% (62.7% vs. 72.9% statin eligible, *p* 0.001). Conclusion: In stable chest pain patients, integration of CAD into guideline recommendations was associated with greater accuracy to reclassify those at increased risk for incident events and a more efficient use of statins.

## 1. Introduction

For decades, scientific evidence has been translated into recommendations for preventive statin treatment to improve cardiovascular health. While primary prevention focuses on an at-risk population before the occurrence of clinical atherosclerotic cardiovascular disease (ASCVD), secondary prevention aims to reduce its impact [1]. However, differentiation between primary and secondary prevention—and thus statin eligibility—may be difficult as symptoms can be unspecific. For instance, in the anatomic testing arm of the Prospective Multicenter Imaging Study for Evaluation of Chest Pain (PROMISE) trial, a cohort of patients with stable chest pain, events occurred in only 3.3% [2]. This is similar to event rates in primary prevention cohorts without known ASCVD [3,4,5].

Coronary Computed Tomography Angiography (CTA) is a relatively new modality that provides a nuanced evaluation of extent and severity of coronary artery disease (CAD). While the vast majority of new-onset stable chest pain patients do not have obstructive CAD as the etiology of their chest pain, many do have varying degrees of subclinical disease making them subject to prevention guidelines. Given the strong relationship between extent and severity of coronary CTA-detected CAD and prognosis [4,5], it would be intuitive to further risk stratification by incorporating coronary CTA-detected CAD assessment into decision-making regarding cardiovascular preventive therapies such as statin initiation.

Hence, in this analysis, we sought to identify an approach based on both current guideline recommendations and coronary CTA to better target cardiovascular preventive therapies to those who will benefit most, and potentially avoid drug-associated side effects in those who may not stand to benefit. We determined the accuracy of the 2018 ACC/AHA guidelines eligibility criteria for statin therapy to identify participants in the PROMISE trial at higher risk for having coronary CTA-detected CAD and for developing cardiovascular events. Furthermore, we sought to integrate the presence of CAD as detected by coronary CTA into the pooled cohort equation (PCE) and determine whether modification of PCE results in reclassification of statin therapy recommendation in the PROMISE cohort.

## 2. Methods

### 2.1. Study Design and Population

The PROMISE trial (URL: http://clinicaltrials.gov. Unique identifier: NCT01174550) was a pragmatic comparative effectiveness trial that enrolled 10,003 patients at 193 enrolling sites in North America, representing both community practices and academic medical centers. Details regarding the PROMISE study population, selection criteria, design and primary results have been described elsewhere [2,6]. Briefly, the study participants were stable symptomatic outpatients without known CAD who required noninvasive cardiovascular testing for further evaluation.

**Figure 1:**
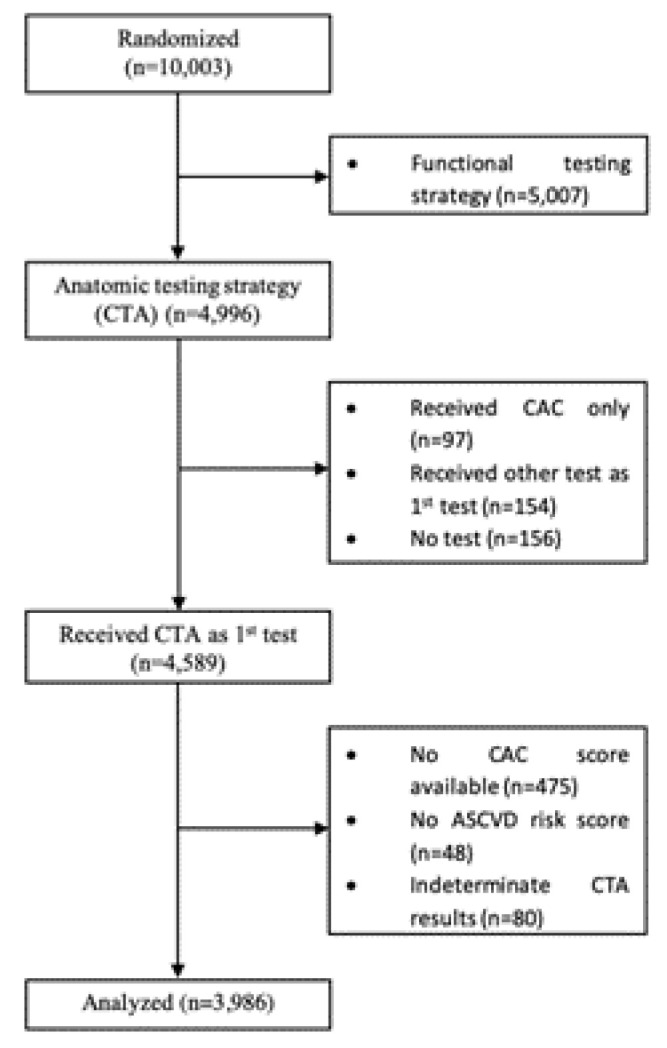
Patient Selection. CTA: Computed Tomography Angiography, CAC: Coronary artery calcium.

For this analysis, we included patients who were randomized to CTA as the initial diagnostic test (including a non-contrast scan for coronary artery calcium (CAC) scoring) and had sufficient risk factor data to assess guideline-based statin eligibility. We excluded subjects who received other tests as their first test, did not undergo CTA or received non-contrast CTA only. The selection of patients for this analysis is detailed in Figure 1.

### 2.2. Study Procedures and Coronary CTA

All included participants provided written informed consent and were randomly assigned to the CTA group. Enrollment began on 27 July 2010 and was completed on 19 September 2013. CTA was performed and interpreted by local physicians who made all subsequent clinical decisions. Appropriate medical therapy was encouraged, and guideline-based educational materials were provided to patients and providers. Follow-up visits were performed at 60 days at the study sites and centrally by means of telephone or mail at 6-month intervals after randomization, for a minimum of 1 year until 31 October 2014. Diagnostic testing was performed in compliance with professional society guidelines. CAC score was assessed according to the Agatston method [7]. Coronary CTA was performed with at least 64-slice multidetector CT technology. Site-reported test results were prospectively classified as no, non-obstructive (at least one diameter stenosis of 1–69%) or obstructive (at least one diameter stenosis of ≥70%) CAD.

### 2.3. Guideline-Based Statin Eligibility

Patient demographics and traditional cardiovascular risk factors were assessed and documented in a standard fashion at the time of enrollment into the PROMISE trial. Per the 2018 ACC/AHA guidelines on the Treatment of Blood Cholesterol to Reduce Atherosclerotic Cardiovascular Risk in Adults [1], we identified candidates for statin treatment based on four delineated benefit groups outlined in the document [1]: clinical atherosclerotic cardiovascular disease (ASCVD), defined as acute coronary syndrome, history of myocardial infarction, stable or unstable angina or other arterial revascularization, stroke, transient ischemic attack or peripheral artery disease [2]; LDL ≥ 190 mg/dL and 20–75 years [3]; diabetes and ≥40 years [4] or without clinical ASCVD or diabetes; LDL 70–189 mg/dL; and estimated ASCVD risk ≥ 5% (based on optional threshold outlined in the guidelines) and individual CAC score >0. ASCVD risk was determined using the pooled cohort calculator [1].

### 2.4. Revision of ASCVD Risk and Modified Statin Eligibility Incorporating Coronary CTA-Detected CAD

Hazard ratios (HR) of non-obstructive and obstructive CAD for events (with no CAD as baseline) were determined using Cox proportional hazard models using this PROMISE cohort data. Using the method described by Emami et al. [8], we incorporated these HR into the ACC/AHA PCE and calculated a revised ASCVD risk and assessed re-classification of statin eligibility [8]. In short, the 10-year risk for ASCVD was derived from the pooled cohort equation as described by Goff et al. [9] To revise gender- and ethnicity-based ASCVD risk thresholds in consideration of the presence or absence of non-obstructive or obstructive CAD, we incorporated the pooled log-HR of non-obstructive and obstructive CAD for ASCVD events from PROMISE into the original equation.

### 2.5. Outcomes

The primary outcome was a composite of death, myocardial infarction or unstable angina over median follow-up of 26 months. Additional composite endpoints analyzed included [1] cardiovascular death, myocardial infarction or unstable angina and [2] cardiovascular death or myocardial infarction. An independent clinical events committee adjudicated all end point events in a blinded fashion on the basis of standard, prospectively determined definitions.

### 2.6. Statistical Analysis

Continuous variables are presented as mean ± standard deviation or median (25th–75th percentile). Categorical variables are presented as absolute and relative frequencies. Comparisons between groups were performed with the use of a two-sample Student t-test or Wilcoxon rank-sum test for continuous variables and Fisher exact test for categorical variables. McNemar’s test was used for comparisons between statin eligibility criteria. The Cox proportional hazards model was used to assess the relationship of statin eligibility to time to the first clinical event (or censoring) for the composite endpoint. Based on these regression models Harrell’s C (C-statistic) was calculated. Kaplan-Meier curves of the cumulative event rates were presented by statin eligibility status and compared using the log rank test [10,11]. A two-sided *p* value of ≤0.05 was considered significant. Analyses were performed with the use of Stata software version 14.2 (StataCorp LP, College Station, Texas)

## 3. Results

### 3.1. Study Population

Overall, 80% of patients from the CTA arm of the PROMISE trial (*n*= 3986/4996) were included in this analysis. As detailed in Figure 1, patients were excluded from the CTA arm if they received only CAC testing (*n* = 97), another initial test (*n* = 154) or did not undergo CTA (*n* = 156). Of those that underwent CTA as initial test (*n* = 4589), 475 were excluded because no CAC score was available, 48 because of missing information on ASCVD risk and 80 for indeterminate CTA result.

Among the 3986 patients with complete data (60.5 ± 8.2 years; 51% female), 67.2% (2678/3986) had non-obstructive or obstructive CAD and 72.9% (2904/3986) were statin eligible. The demographics, cardiovascular risk factors and cardiovascular event rates stratified by guideline-based statin eligibility status are detailed in Table 1. Those who were statin-eligible were slightly older (mean age 60.9 vs. 59.5 years), more likely to be male (54.8% vs. 31.9%) and had a greater prevalence of traditional cardiovascular risk factors including hypertension (67.3% vs. 56.5%), diabetes (27.8% vs. 0.0%) and tobacco use (61.3% vs. 23.6%) than those who were not statin eligible (*p* < 0.001 for all). Mean LDL of statin-eligible patients was 116.2 mg/dL versus 115.7 mg/dL for statin-ineligible patients (*p* = 0.530). Nearly half of patients were on a statin at baseline, with a higher proportion in statin-eligible patients than in statin-ineligible patients (48.0% vs. 39.4%, *p* < 0.001).

### 3.2. Guideline-Based Statin Eligibility and Cardiovascular Events

**Figure 2:**
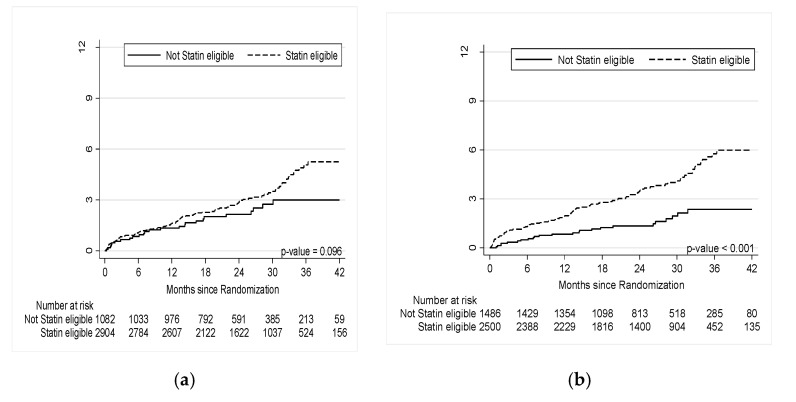
Kaplan–Meier curves for primary endpoints (all-cause death/MI/UA) in statin eligible vs. not eligible: (**a**) according to current 2018 Guidelines including CAC; and (**b**) using the modified 2018 Guidelines including CAC and modified CAD score.

During a median follow-up of 26 months, there were 122 (3.1%) events (57 death, 20 myocardial infarction and 47 unstable angina). Statin-eligible patients had a 3.3% event rate versus 2.3% for statin ineligible (HR = 1.4 (95% CI 0.9–2.3), *p* = 0.098). (Figure 2a and Table 2a). For the additional composite endpoint of cardiovascular death, myocardial infarction or unstable angina, statin-eligible patients had a 2.6% event rate versus 1.9% for statin ineligible (HR = 1.3 (95% CI 0.8–2.2), *p* = 0.239). For the composite endpoint of cardiovascular death or myocardial infarction, statin-eligible patients had a 1.4% event rate versus 1.0% for statin ineligible (HR = 1.4 (95% CI 0.7–2.7), *p* = 0.367).

Primary event rates increased with increasing severity of CAD among both statin-eligible and statin-ineligible patients. However, there was discordance between statin eligibility and CAD, with 15.4% (73/475) of patients with obstructive CAD not eligible and 54.3% (710/1308) without any CAD being statin eligible. Notably, those statin-ineligible patients with obstructive CAD had a similarly high event rate compared to those statin-eligible with obstructive disease (11.0% vs. 9.7%), while statin-eligible patients with no CAD had a comparably low event rate to those with no CAD and no recommendation for statin treatment (1.1% vs. 0.7%) (Table 2a, top).

### 3.3. Modified Statin Eligibility and Cardiovascular Events in all Patients

Utilizing the revised statin eligibility rule, which incorporated information regarding coronary CTA-detected CAD into the PCE (Table 2b, bottom), statin-eligible patients had a 3.9% event rate versus 1.6% for statin ineligible (HR = 2.4 (95% CI 1.6–3.8 *p* < 0.001). Specifically, event rates increased in statin-eligible patients from 3.3% (97/2904) to 3.9% (98/2500) and decreased in statin-ineligible patients from 2.3% (25/1082) to 1.6% (24/1486) when comparing guideline-based vs. modified statin eligibility.

**Figure 3:**
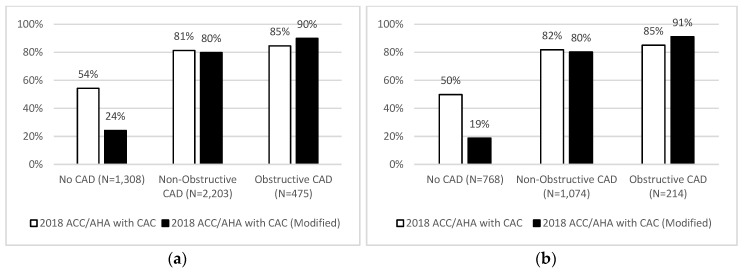
ACC/AHA statin eligibility versus modified statin eligibility stratified by presence and extent of CAD in: (**a**) all patients; and (**b**) statin naïve patients.

As a result, incorporating information on coronary CTA-detected CAD into the PCE reclassified 12.7% of patients. Notably, reclassification towards statin-ineligibility occurred in 12.8% (449/3511) of patients with no CAD or nonobstructive disease (majority with no CAD) while event rates showed a better discrimination (2.3% in statin-eligible vs. 1.7% in statin-ineligible before and 2.6% in statin-eligible vs. 1.5% in statin-ineligible patients after incorporating information on coronary CTA-detected CAD). Specifically, in all patients with non-obstructive CAD, net-reclassification towards statin ineligibility occurred in 1.6% (35/2203). (Figure 2a and Figure 3a) This improved discrimination for events (c statistic: 0.59 (CI 95%: 0.55–0.62) vs. 0.52 (CI 95%: 0.49–0.56), *p* < 0.001) while reducing statin use (62.7% (2500/3986) vs. 72.9% (2904/3986) statin eligible, *p* < 0.001).

### 3.4. Stratified Analysis of Modified Statin Eligibility and Cardiovascular Events in Statin Naïve Patients

Similar results were found in a stratified analysis of the revised statin eligibility rule in 2074 statin naïve patients (Table 2b, bottom). Statin-eligible patients had a 4.0% event rate versus 2.1% for statin ineligible (HR = 1.97 (95% CI 1.14–3.38); *p* = 0.014). Event rates increased in statin-eligible patients from 3.4% (49/1451) to 4.0% (48/1203) and decreased in statin-ineligible patients from 2.7% (17/623) to 2.1% (18/871) when comparing guideline-based and modified statin eligibility. This resulted in reclassification of a total of 13.9% of statin naïve patients, the majority of which had no or non-obstructive CAD and was reclassified towards no-statin (14.4% (268/1860)). Specifically, in statin naïve patients with non-obstructive CAD, net-reclassification towards statin ineligibility occurred in 1.6% (17/1074) (Figure 2b and Figure 3b).

## 4. Discussion

The presence of coronary CTA-detected CAD, a common finding in patients with new onset, stable chest pain, carries a higher risk for incident ASCVD events as compared to no CAD [4]. In this study, we demonstrated that incorporation of this information into the ASCVD risk calculator results in modification of ASCVD risk and favorable reclassification of statin eligibility, with improved discrimination for cardiovascular events. Use of the reclassified risk would substantially reduce overall statin prescription among those without CAD, while similar proportions of those with non-obstructive and obstructive CAD would be treated. For example, *in those without coronary CTA-detected CAD*, the rate of statin eligibility was reduced by 30% by incorporating this information into the PCE, with similar overall downstream cardiovascular event rates (1.1% for statin-eligible patients using original ASCVD guideline versus 1.3% with modified statin eligibility).

Coronary CTA-guided “downgrading” of statin eligibility is still a topic in its infancy. However, our study building on primary prevention data showed that the absence of coronary artery calcification identifies a large group (33%) of statin eligible individuals who are at a similarly low risk as non-statin eligible individuals (1.0% and 1.1% 10-year ASCVD risk, respectively) [12]. While CAC scoring as recommended by the 2018 AHA/ACC primary prevention guidelines for certain intermediate risk patients is an additional “screening test”, symptomatic chest pain patients undergo coronary CTA as part of the workup to evaluate chest pain. Therefore, the use of coronary CTA findings in symptomatic patients to individualize statin eligibility decisions comes without any intrinsic additional cost. Moreover, CTA-based assessment of CAD includes information on the presence of CAC as well as non-calcified and high-risk plaques. In a population of patients with suspected CAD, at least one plaque with ≥50% stenosis was found in 19% of patients with a CAC score of 0 [11]. This carries significant prognostic information for adverse cardiac events [11,13,14]. One of the reasons why it has been proven of incremental to traditional ASCVD risk and the CAC score for the prediction of events (C-statistic 0.776 vs. 0.682; *p* < 0.001) [15].

Our finding may be important as concerns were raised that the new ASCVD risk score recommends treatment that would results in a substantial increase in the adult population eligible for statin therapy, from 43 to 56 million adults of U.S. adults between age 40 and 75 years eligible for statin therapy because the vast majority of re-classification occurred towards new statin eligibility as compared to the ATP-III guidelines [16]. This concept of “downgrading” statin eligibility may also may be important for younger patients being considered for long-term statin therapy and individuals who may be at higher risk for adverse effects [17]. However, to prove the concept of CTA-guided “downgrading”, future randomized clinical trials will be needed.

In clinical practice, few would not consider a patient with obstructive CAD to be eligible for statin therapy, obviating the need for reclassification of statin eligibility in this case. However, in patients with non-obstructive CAD, there is a lack of consensus on whether these patients need be treated with preventive statin therapy. However, it is known that statins can slow plaque progression or even reduce atheroma burden [18,19]. Moreover, there is evidence suggesting mortality reduction in patients with non-obstructive CAD on baseline statin treatment [20,21]. This analysis provides additional evidence that incorporating information regarding the presence of non-obstructive CAD into statin eligibility decision-making leads to overall better accuracy in identifying those who develop cardiovascular events.

A limitation of this study, which may provide further nuanced management particularly in the non-obstructive CAD patients, is that advanced coronary CTA coronary plaque assessments are not incorporated into the risk assessment. More diffuse non-obstructive CAD (i.e., high SYNTAX score), presence of high-risk plaque features and identification of hemodynamically significant lesions (using CT-FFR evaluation) may optimize identification of non-obstructive CAD patients at risk for events [22,23] a subgroup that could potentially be “upgraded” to statin eligibility. Conversely, absence of these features may result in “downgrading” to statin ineligible. Moreover, the median follow-up of 26 months is rather short, especially with regards to the PCE that are intended to predict 10-year ASCVD events. While this may limit the generalizability of our results, CTA-based “downgrading” is still a theoretical concept which will need further investigation in prospective randomized trials. It also needs to be mentioned that nearly half of the patients in this analysis were already on a statin. Nevertheless, data from our sub-analysis in statin naïve patients suggest similar outcomes with an even higher percentage of patients without CAD being “downgraded” to a no-statin approach.

Concluding, in this stable cohort with symptoms suggestive of CAD, integration of CAD findings into ACC/AHA guideline determination of statin eligibility was associated with greater accuracy to identify those at increased risk for incident events and resulted in more efficient statin allocation. Consideration should be made to implement this approach in prevention guidelines.

## Figures and Tables

**Table 1:** Patient demographics, cardiovascular risk factors, clinical presentation, risk score, outcomes.

Variables	All Patients (*n* = 3986)	Not Statin Eligible (*n* = 1082)	Statin Eligible (*n* = 2904)	*p*-Value
Age, mean ± SD	60.5 ± 8.2	59.5 ± 9.8	60.9 ± 7.4	0.001
Male, *n* (%)	1936 (48.6)	345 (31.9)	1591 (54.8)	0.001
Race, *n* (%)				
White	3364 (84.4)	929 (85.9)	2435 (83.9)	0.128
Black	407 (10.2)	89 (8.2)	318 (11.0)	0.011
Other	215 (5.4)	64 (5.9)	151 (5.2)	0.386
CV risk factors				
Body-Mass Index (kg/m^2^), mean ± SD	30.3 ± 5.9	29.3 ± 5.8	30.7 ± 5.8	0.001
Systolic Blood Pressure (mmHg), mean ± SD	131.2 ± 16.4	127.9 ± 16.7	132.4 ± 16.2	0.001
Diastolic Blood Pressure (mmHg), mean ± SD	78.9 ± 10.1	77.9 ± 10.6	79.2 ± 10.0	0.001
Hypertension, *n* (%)	2566 (64.4)	611 (56.5)	1955 (67.3)	0.001
Diabetes, *n* (%)	808 (20.3)	0 (0.0)	808 (27.8)	0.001
Dyslipidemia, *n* (%)	2693 (67.6)	698 (64.5)	1995 (68.7)	0.013
Family History of Premature CAD, No./Total No. (%)	1307/3976 (32.9)	277/1079 (25.7)	1030/2897 (35.6)	0.001
Peripheral Arterial or Cerebrovascular Disease, No./Total No. (%)	208 (5.2)	6 (0.6)	202 (7.0)	0.001
CAD Risk Equivalent, *n* (%)	957 (24.0)	6 (0.6)	951 (32.8)	0.001
Metabolic Syndrome, *n* (%)	1465 (36.8)	203 (18.8)	1262 (43.5)	0.001
Current or Past Tobacco Use, No./Total No. (%)	2036 (51.1)	255 (23.6)	1781 (61.3)	0.001
Sedentary Lifestyle, No./Total No. (%)	1917/3978 (48.2)	503/1079 (53.4)	1414/2899 (48.8)	0.239
History of Depression, No. (%)	766 (19.2)	205 (19.0)	561 (19.3)	0.821
Risk Burden				
No Risk Factors, No. (%)	103 (2.6)	52 (4.8)	51 (1.8)	0.001
Mean No. of Risk Factors Per Patient	2.36 ± 1.08	1.70 ± 0.84	2.61 ± 1.05	0.001
Mean Combined Diamond and Forrester and Coronary Artery Surgery Study Risk Score	53.2 ± 21.2	45.1 ± 20.8	56.2 ± 20.5	0.001
Framingham Risk Score Categories, No./Total No. (%)				0.001
Low Risk (6%)	262 (6.6)	230 (21.3)	32 (1.1)	
Intermediate Risk (6–20%)	2088 (52.4)	732 (67.7)	1356 (46.7)	
High Risk (20%)	1636 (41.0)	120 (11.1)	1516 (52.2)	
Framingham Risk Score, median (IQR)	17.0 (10.5–27.9)	8.8 (6.3–13.3)	20.8 (13.9–31.6)	0.001
ASCVD Risk, No./Total No. (%)				0.001
Low Risk (7.5%)	1302 (32.7)	782 (72.3)	520 (17.9)	
Elevated Risk (≥7.5%)	2684 (67.3)	300 (27.7)	2,384 (82.1)	
ASCVD Risk Median (IQR)	11.0 (6.1–19.2)	4.4 (3.0–8.6)	13.2 (8.5–20.7)	0.001
Lipids, mean ± SD				
Total Cholesterol, mg/dL	195.9 ± 34.5	195.2 ± 29.1	196.1 ± 36.3	0.430
LDL Cholesterol, mg/dL	116.1 ± 27.4	115.7 ± 24.3	116.2 ± 28.4	0.530
HDL Cholesterol, mg/dL	51.4 ± 12.1	55.5 ± 12.8	49.9 ± 11.5	0.001
Triglycerides, mg/dL	155.7 ± 158.4	125.2 ± 65.5	166.5 ± 178.9	0.001
Baseline Medications, No./Total No. (%)				
Beta-Blocker	953/3817 (25.0)	207/1028 (20.1)	746/2789 (26.8)	0.001
ACE Inhibitor or ARB	1655/3817 (43.4)	332/1028 (32.3)	1323/2789 (47.4)	0.001
Statin	1743/3817 (45.7)	405/1028 (39.4)	1338/2789 (48.0)	0.001
Aspirin	1722/3817 (45.1)	395/1028 (38.4)	1327/2789 (47.6)	0.001
Clopidogrel	48/3817 (1.3)	8/1028 (0.8)	40/2789 (1.4)	0.139
Prasugrel	1/3817 (0.03)	0/1028 (0.0)	1/2789 (0.04)	1.000
Warfarin	59/3817 (1.6)	15/1028 (1.5)	44/2789 (1.6)	0.883
Primary Presenting Symptom, No./Total No. (%)				
Chest Pain	2930 (73.5)	817 (75.5)	2113 (72.8)	0.090
Dyspnea on Exertion	569 (14.3)	132 (12.2)	437 (15.1)	0.022
Other	485 (12.2)	133 (12.3)	352 (12.1)	0.913
Type of Angina, No. (%)				0.001
Typical	440 (11.0)	96 (8.9)	344 (11.9)	
Atypical	3125 (78.4)	843 (77.9)	2282 (78.6)	
Nonanginal Pain	421 (10.6)	143 (13.2)	278 (9.6)	
Pretest Likelihood of Significant CAD (Physician Based), No./Total No. (%)				0.001
Very Low (10%)	228/3980 (5.7)	76/1080 (7.0)	152/2900 (5.2)	
Low (10–30%)	1228/3980 (30.9)	418/1080 (38.7)	810/2900 (27.9)	
Intermediate (31–70%)	2335/3980 (58.7)	562/1080 (52.0)	1773/2900 (61.1)	
High (71–90%)	174/3980 (4.4)	21/1080 (1.9)	153/2900 (5.3)	
Very High (90%)	15/3980 (0.4)	3/1080 (0.3)	12/2900 (0.4)	
MACE, No. (%)				
Death, Non-Fatal MI or Hospitalization For UAP	122 (3.1)	25 (2.3)	97 (3.3)	
CV Death, Non-Fatal MI or Hospitalization For UAP	96 (2.4)	21 (1.9)	75 (2.6)	
CV Death or Non-Fatal MI	51 (1.3)	11 (1.0)	40 (1.4)	
Non-Fatal MI	20 (0.5)	5 (0.5)	15 (0.5)	

**Table:** (**a**)

Coronary CTA	Total	No CAD	Non-obstructive CAD	Obstructive CAD
**ALL PATIENTS**	*n* = 3986 Event Rate 3.1%	*n* = 1308 Event Rate 0.9%	*n* = 2203 Event Rate 2.9%	*n* = 475 Event Rate 9.9%
Statin eligibility by 2018 ACC/AHA Guidelines with CAC				
**STATIN ELIGIBLE**	*n* = 2904 (72.9) Event Rate 3.3%	*n* = 710 (54.3) Event Rate 1.1%	*n* = 1792 (81.3) Event Rate 2.8%	*n* = 402 (84.6) Event Rate 9.7%
**NOT STATIN ELIGIBLE**	*n* = 1082 (27.2) Event Rate 2.3%	*n* = 598 (45.7) Event Rate 0.7%	*n* = 411 (18.7) Event Rate 3.2%	*n* = 73 (15.4) Event Rate 11.0%
Statin Eligibility by 2018 ACC/AHA Guidelines with CAC incorporating coronary CTA-detected CAD				
**STATIN ELIGIBLE**	*n* = 2500 (62.7) Event Rate 3.9%	*n* = 316 (24.2) Event Rate 1.3%	*n* = 1757 (79.8) Event Rate 2.9%	*n* = 427 (89.9) Event Rate 10.3%
**NOT STATIN ELIGIBLE**	*n* = 1486 (37.3) Event Rate 1.6%	*n* = 992 (75.8) Event Rate 0.8%	*n* = 446 (20.3) Event Rate 2.9%	*n* = 48 (10.1) Event Rate 6.3%

**Table:** (**b**)

Coronary CTA	Total	No CAD	Non-obstructive CAD	Obstructive CAD
**STATIN NAIVE PATIENTS**	*n* = 2074 Event Rate 3.2%	*n* = 786 Event Rate 1.2%	*n* = 1074 Event Rate 3.1%	*n* = 214 Event Rate 11.2%
Statin eligibility by 2018 ACC/AHA Guidelines with CAC				
**STATIN ELIGIBLE**	*n* = 1451 (70.0) Event Rate 3.4%	*n* = 391 (49.8) Event Rate 1.3%	*n* = 878 (81.8) Event Rate 2.7%	*n* = 182 (85.1) Event Rate 11.0%
**NOT STATIN ELIGIBLE**	*n* = 623 (30.0) Event Rate 2.7%	*n* = 395 (50.3) Event Rate 1.0%	*n* = 196 (18.3) Event Rate 4.6%	*n* = 32 (15.0) Event Rate 12.5%
Statin Eligibility by 2018 ACC/AHA Guidelines with CAC incorporating coronary CTA-detected CAD				
**STATIN ELIGIBLE**	*n* = 1203 (58.0) Event Rate 4.0%	*n* = 147 (18.7) Event Rate 1.4%	*n* = 861 (80.2) Event Rate 2.8%	*n* = 195 (91.1) Event Rate 11.3%
**NOT STATIN ELIGIBLE**	*n* = 871 (42.0) Event Rate 2.1%	*n* = 639 (81.3) Event Rate 1.1%	*n* = 213 (19.8) Event Rate 4.2%	*n* = 19 (8.9) Event Rate 10.5%

## Abbreviations

ACS	Acute Coronary Syndrome
ASCVD	Atherosclerotic Cardiovascular Disease
CAD	Coronary Artery Disease
CTA	Ct Angiography
CV	Cardiovascular
PCE	Pooled Cohort Equation
